# Supplementary material for: Exosomes from adipose-derived stem cells alleviate premature ovarian failure via blockage of autophagy and AMPK/mTOR pathway
Source: PeerJ. 2023 Dec 14;11:e16517. doi: 10.7717/peerj.16517 (PMC10725676; doi:10.7717/peerj.16517)
Supplement: Data S1 [file peerj-11-16517-s001.zip › raw data/Figure 4.pdf]

A

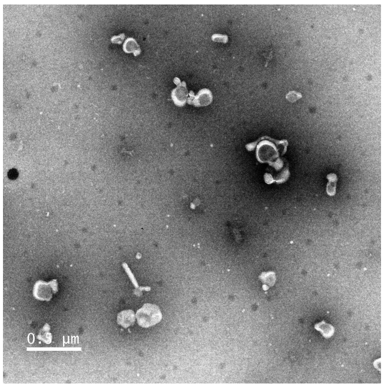

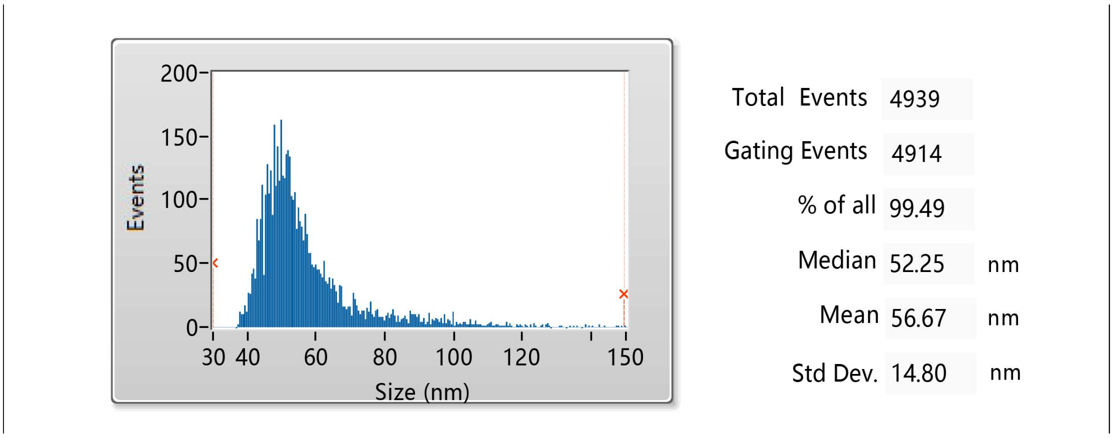
Events
Size (nm)
200
150
100
50
0
30
40
60
80
100
120
150
Total Events 4939
Gating Events 4914
% of all 99.49
Median 52.25 nm
Mean 56.67 nm
Std Dev. 14.80 nm

B

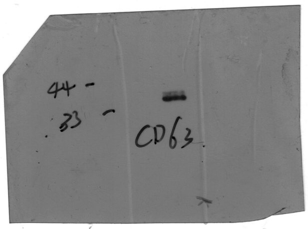
44
33
CD63

C

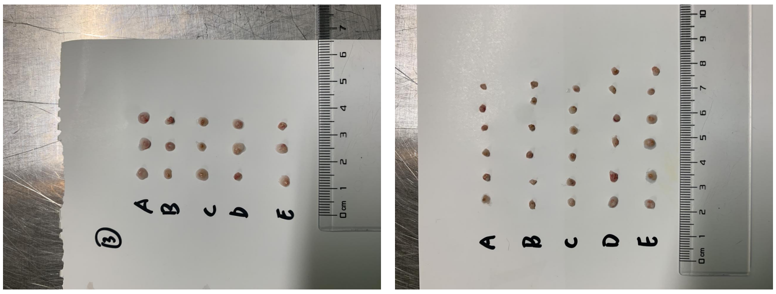
A
B
C
D
E

D

7d

WY

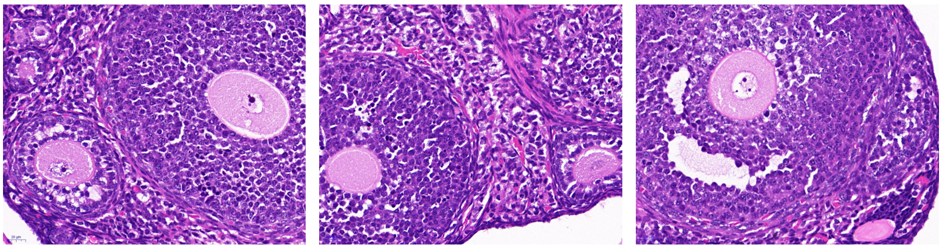

POF

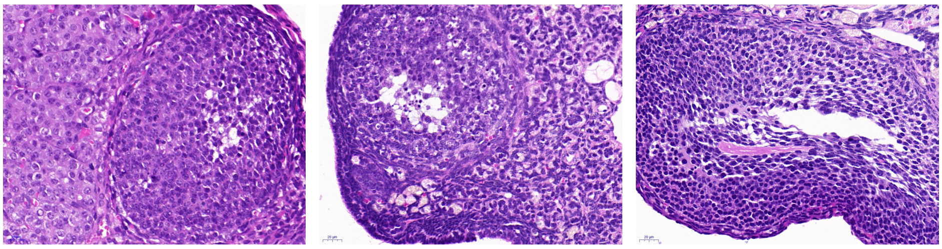

PBS

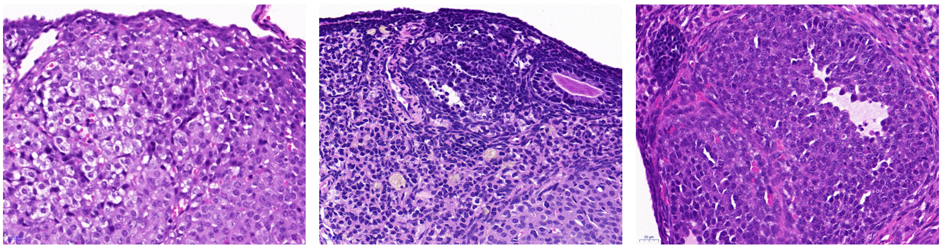

ADSCs

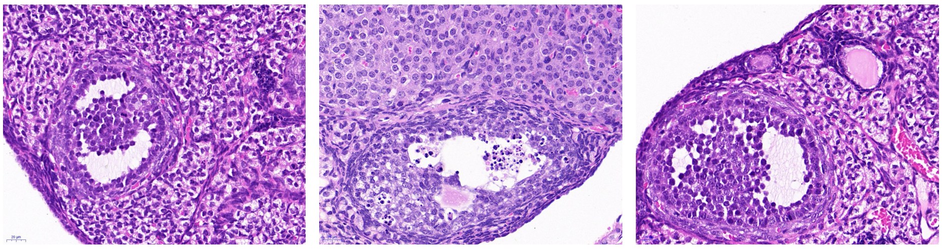

Exo

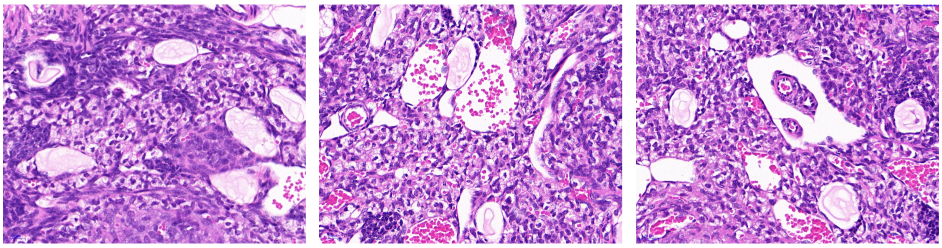

15d

WY

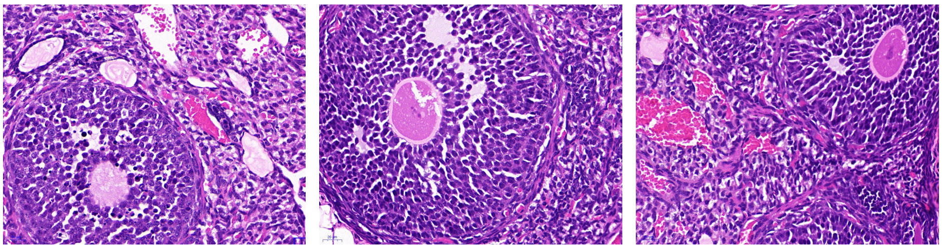

POF

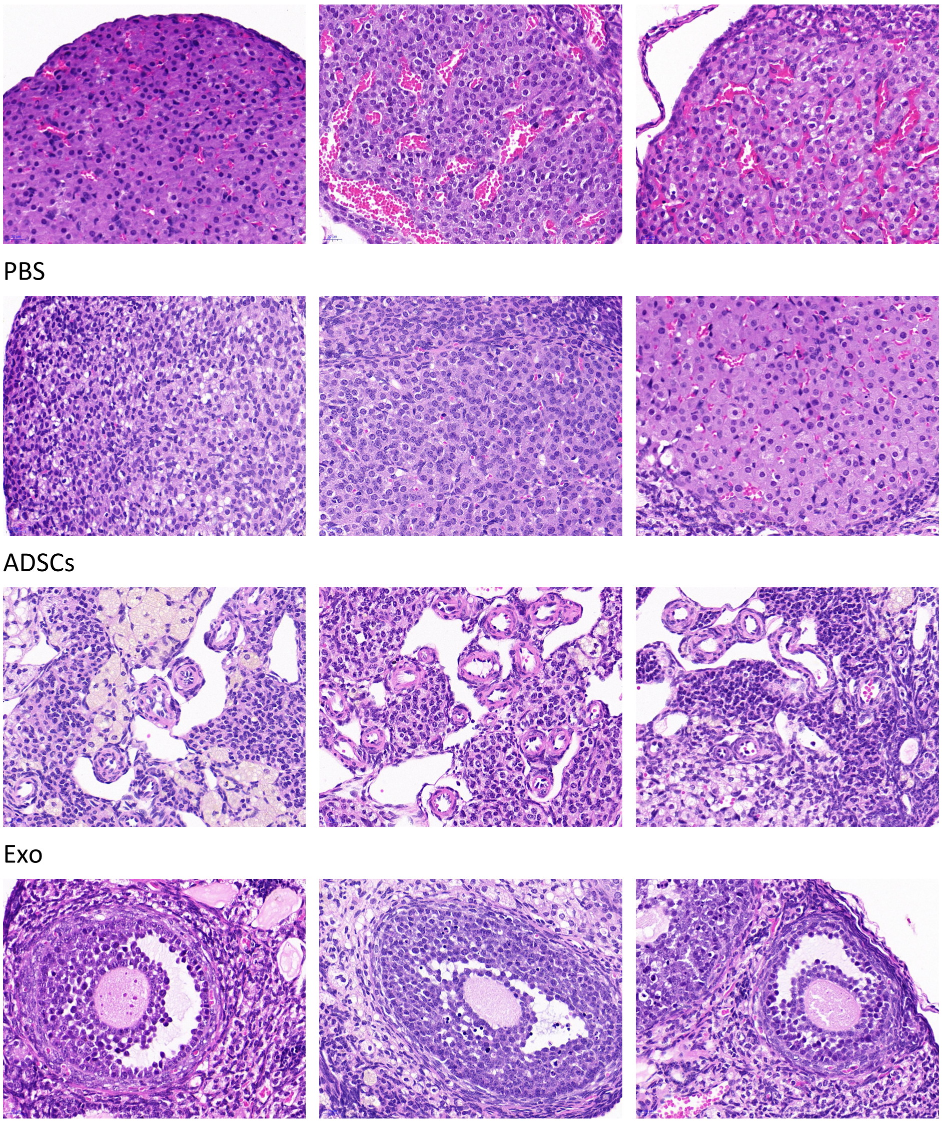
PBS
ADSCs
Exo
