# Supplementary figures and images for: Exosomes from adipose-derived stem cells alleviate premature ovarian failure via blockage of autophagy and AMPK/mTOR pathway
Source: PeerJ. 2023 Dec 14;11:e16517. doi: 10.7717/peerj.16517 (PMC10725676; doi:10.7717/peerj.16517)

A

7d

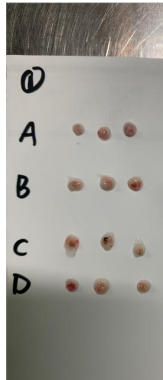

15d

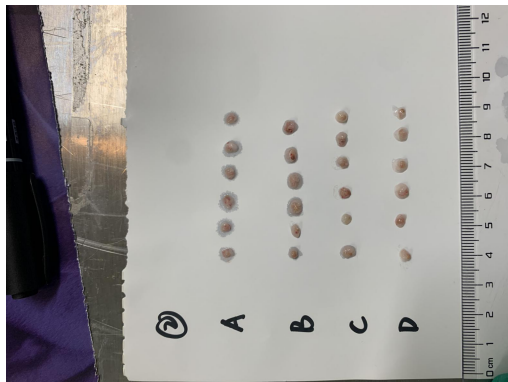

B

7d

WT

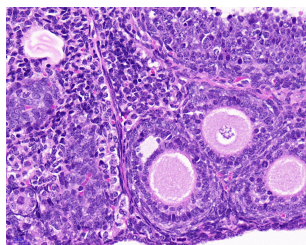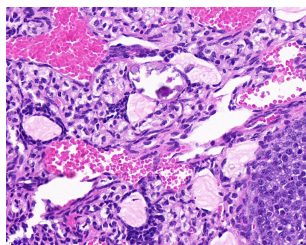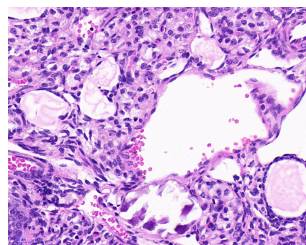

POF

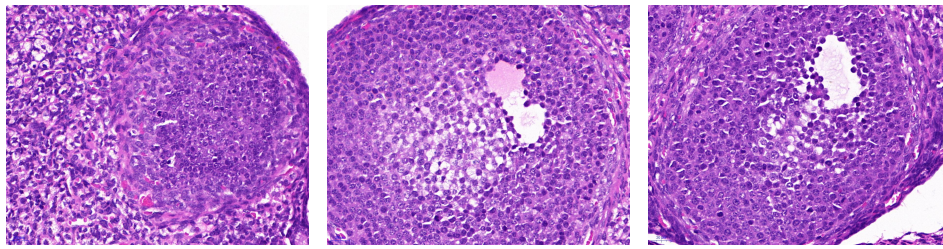

DMSO

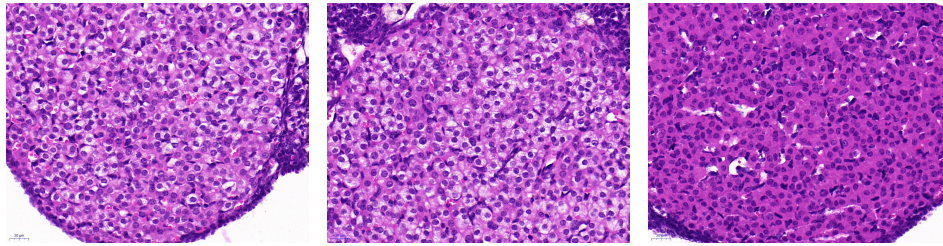

3-MA

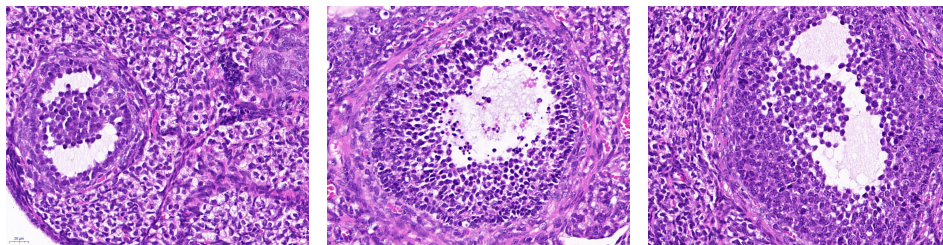

15d

WT

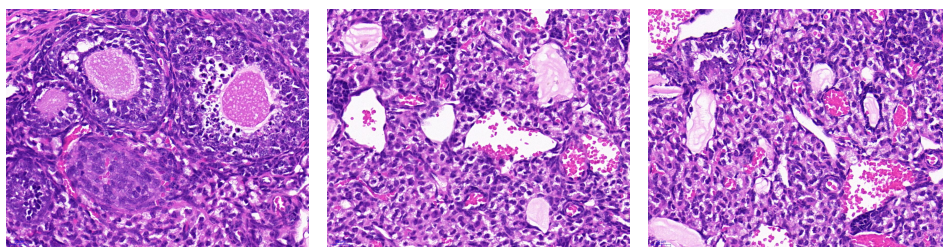

POF

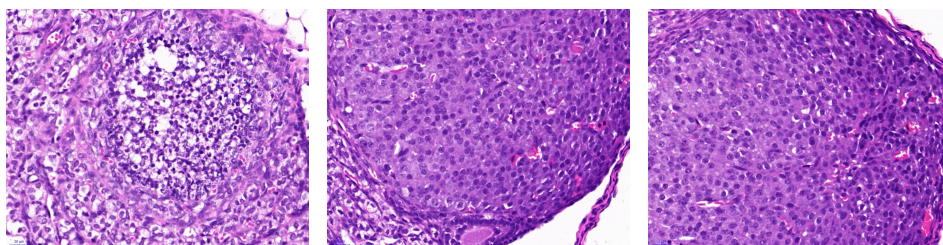

DMSO

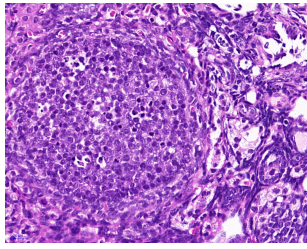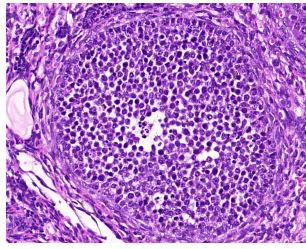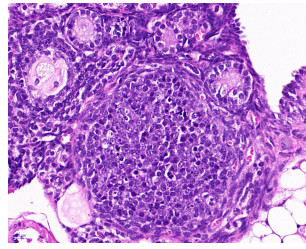

3-MA

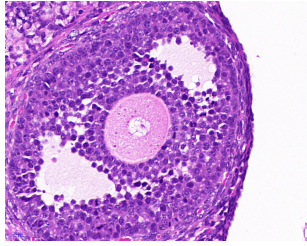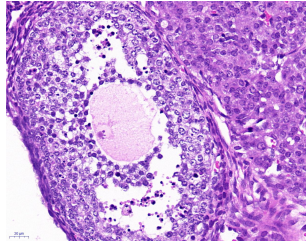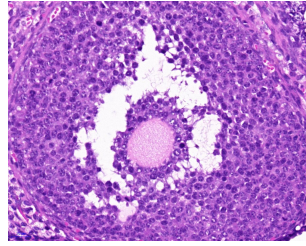

Supplement: Data S1 [file peerj-11-16517-s001.zip › raw data/Figure 1.pdf]

A  
WT

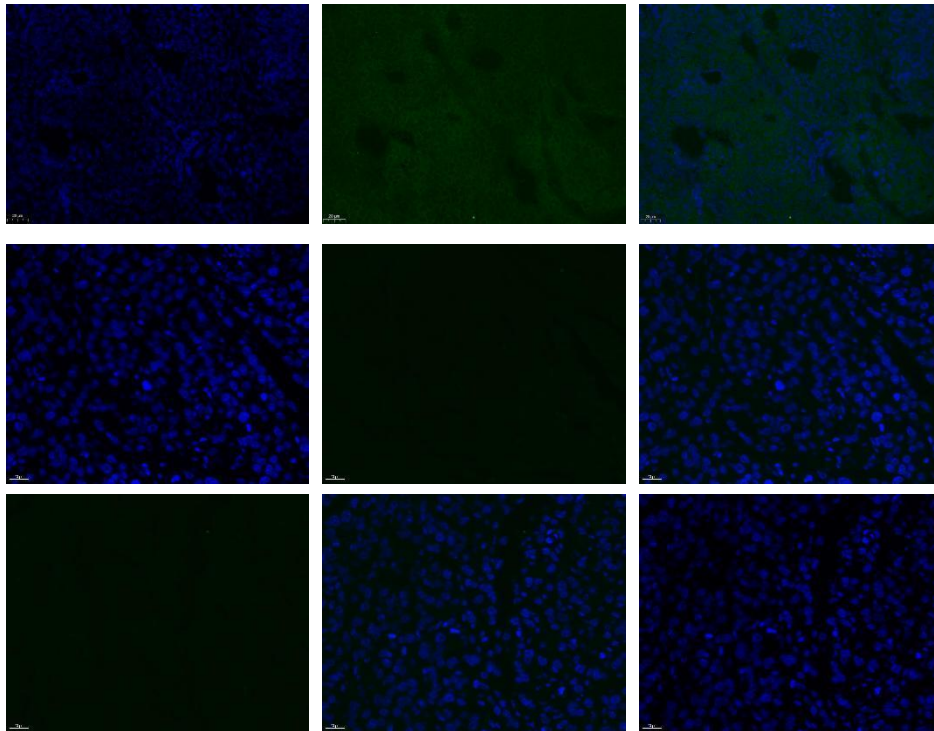

POF

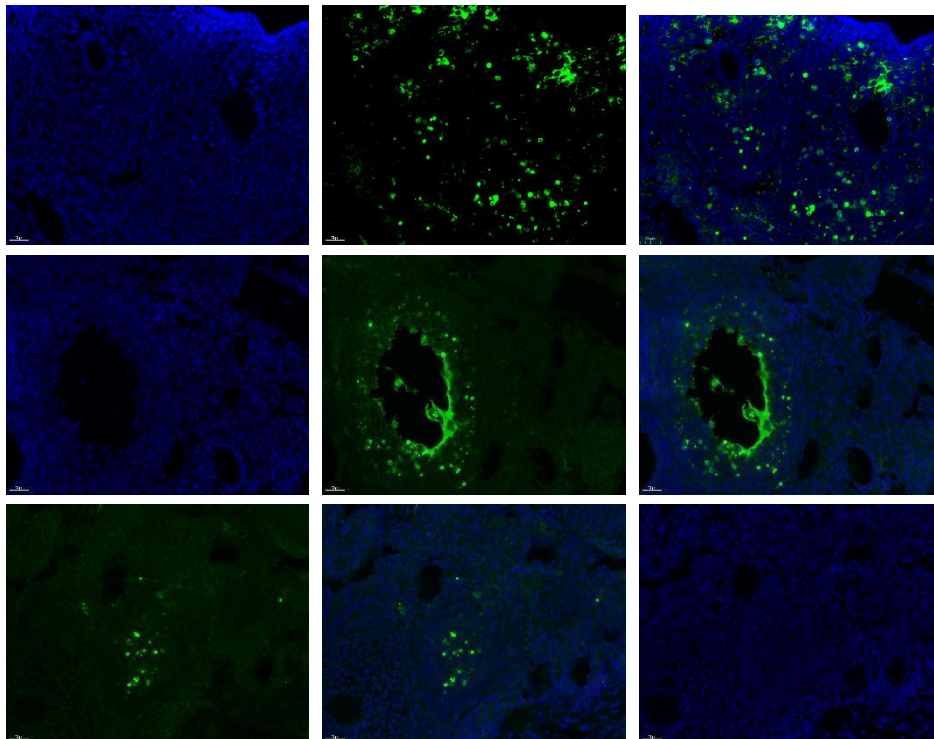

DMSO

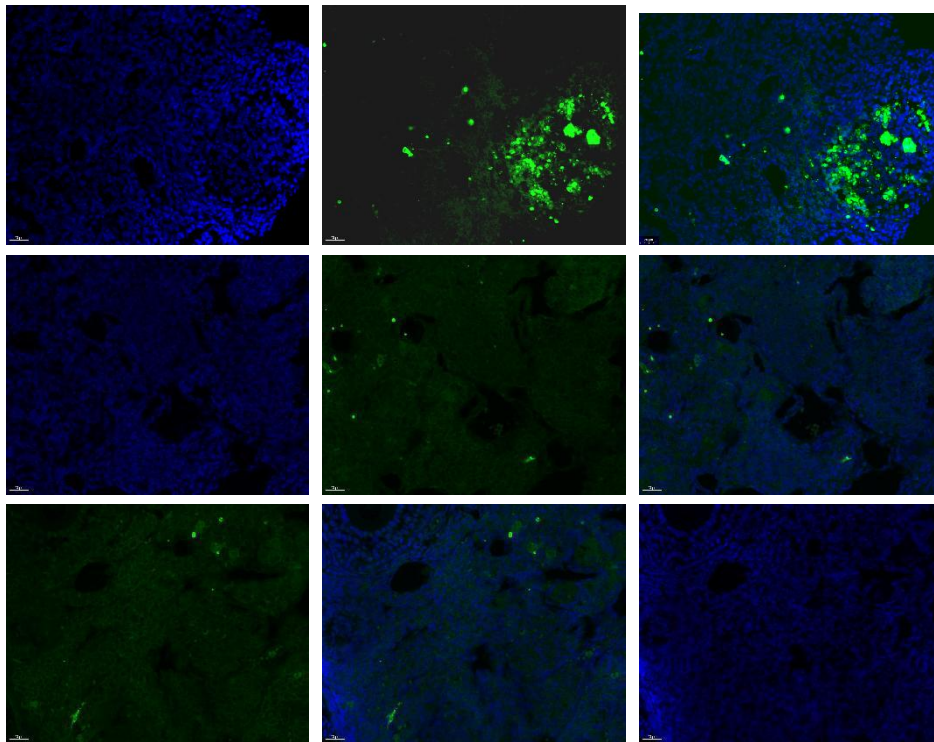

3-MA

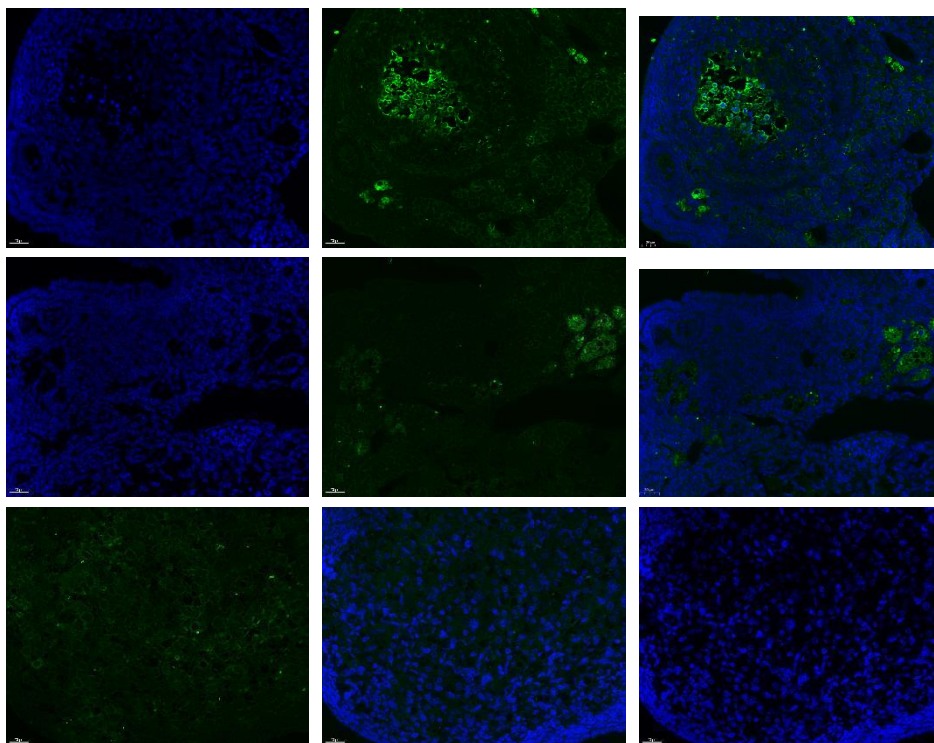

B  
WT

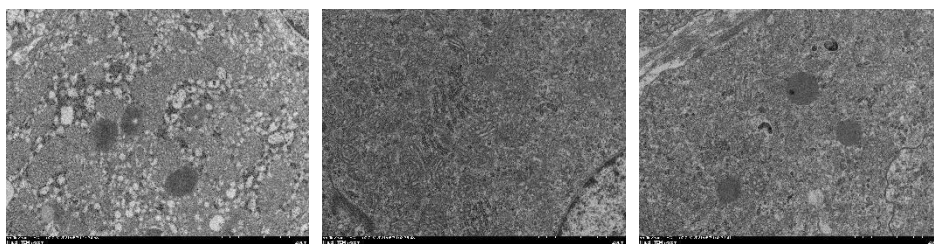

POF

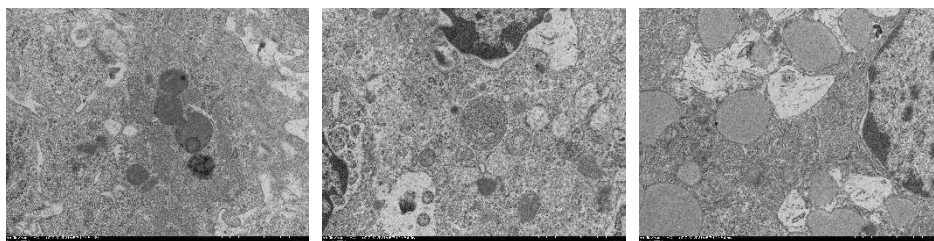

DMSO

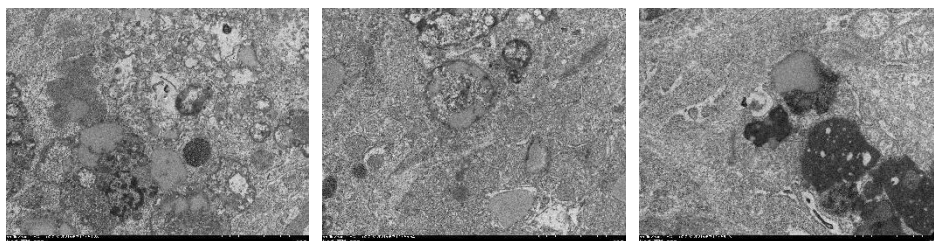

3-MA

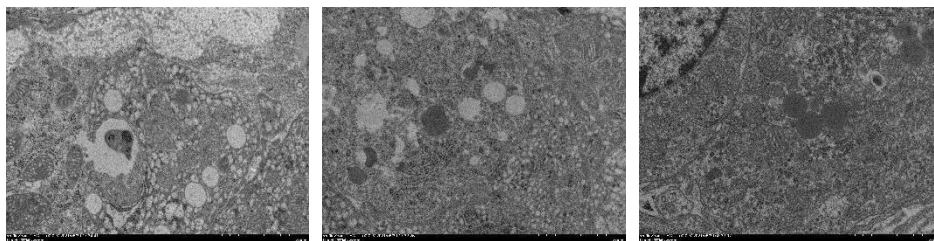

Supplement: Data S1 [file peerj-11-16517-s001.zip › raw data/Figure 2.pdf]

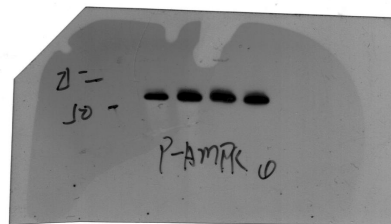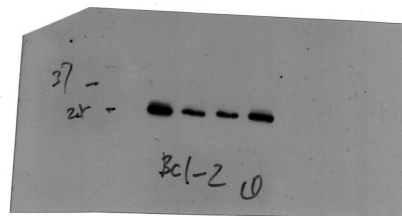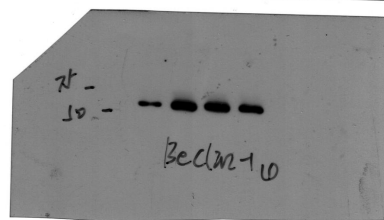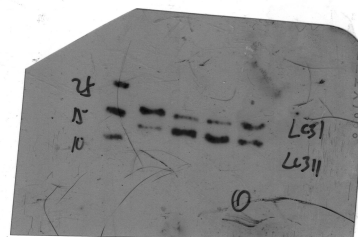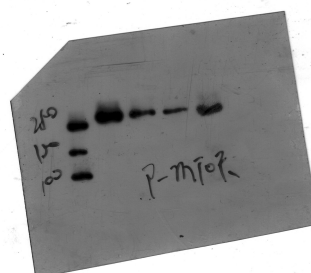

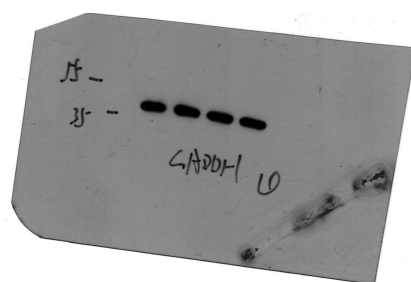

Supplement: Data S1 [file peerj-11-16517-s001.zip › raw data/Figure 3.pdf]

B  
WT

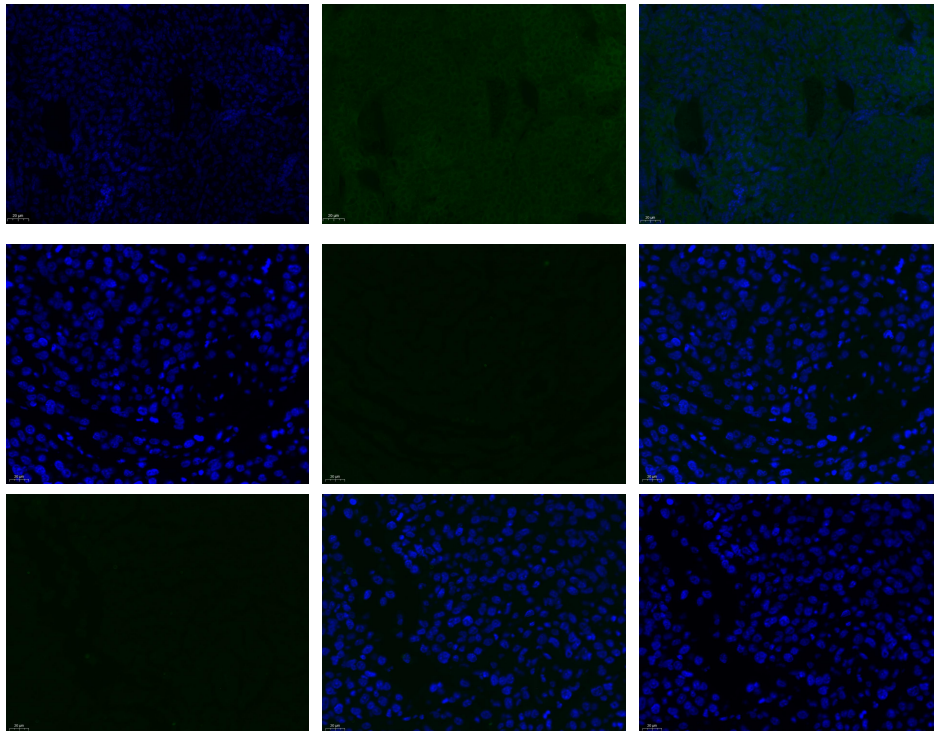

POF

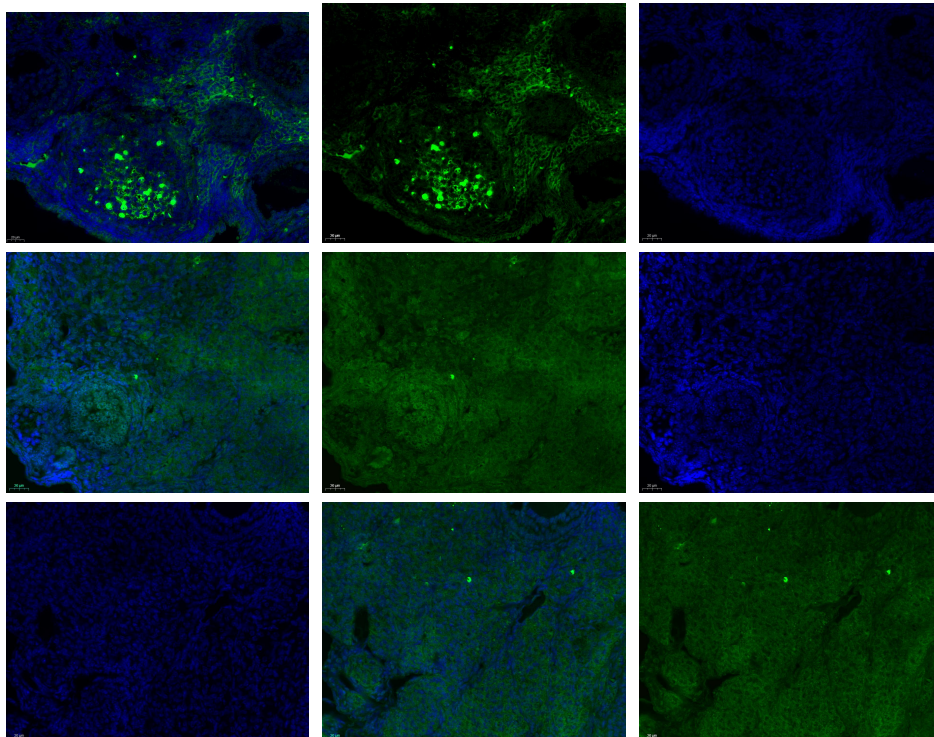

PBS

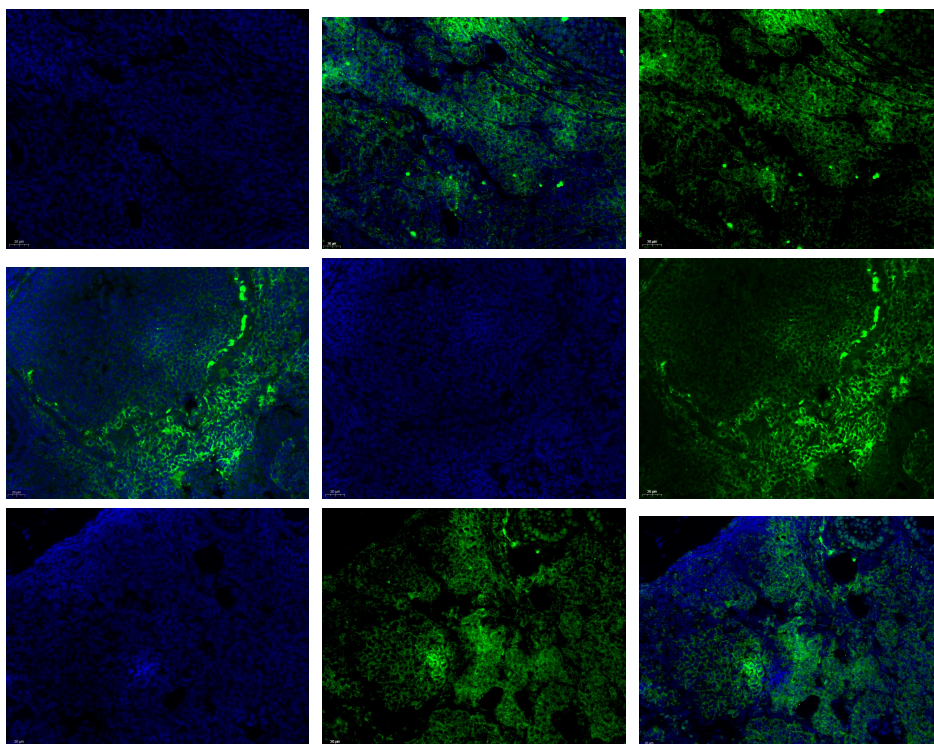

ADSCs

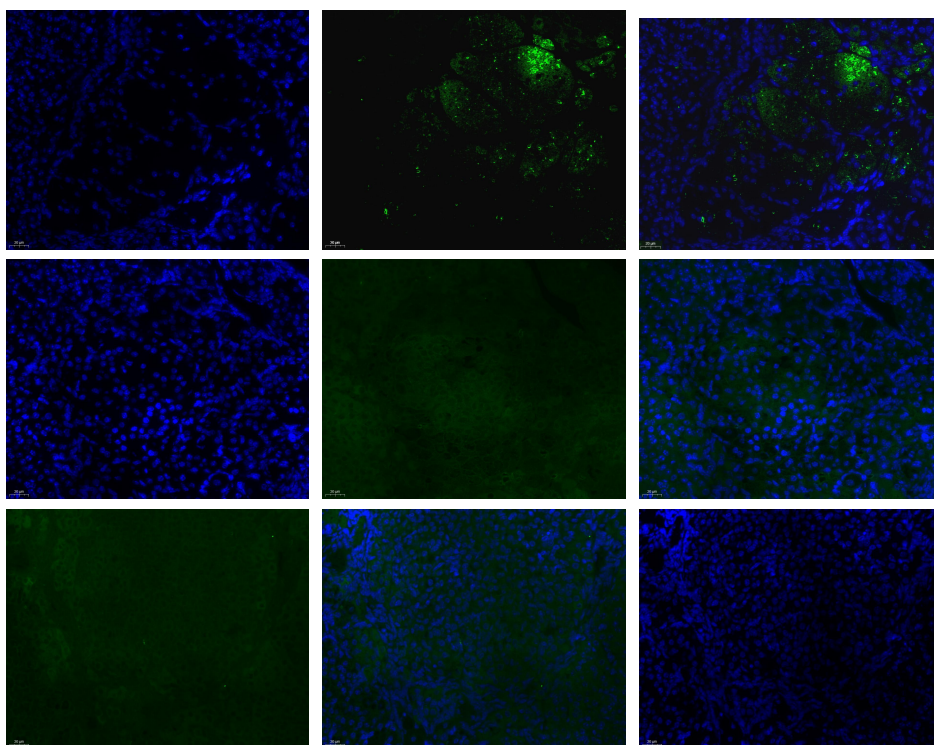

Exo

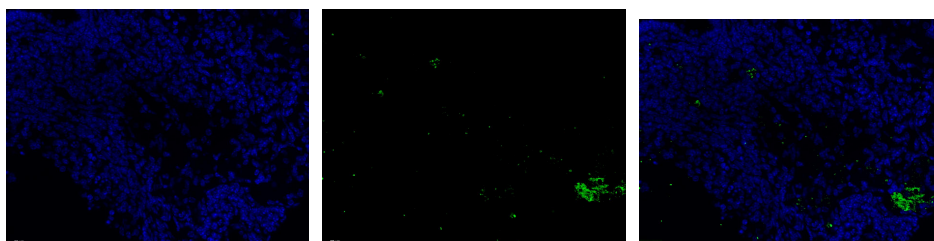

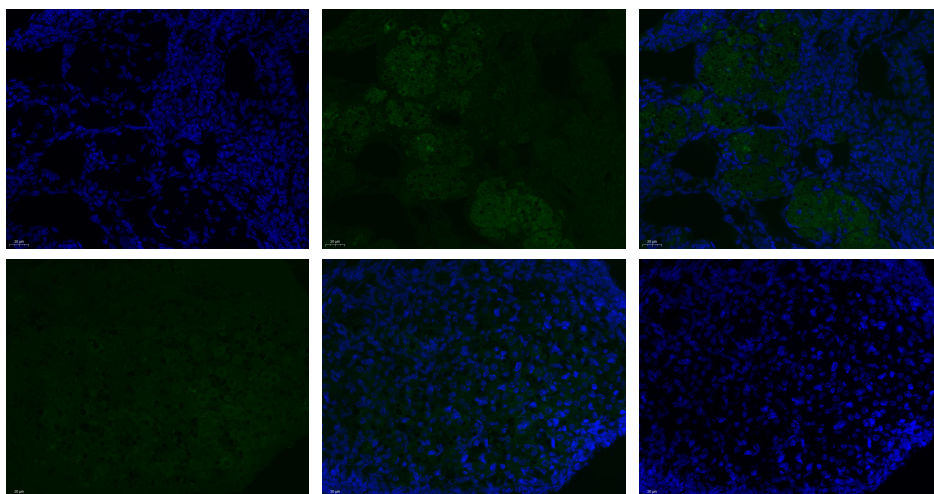

C

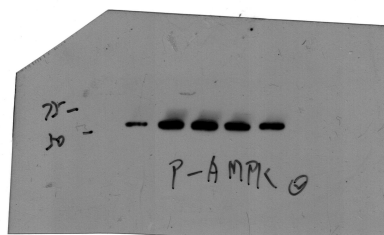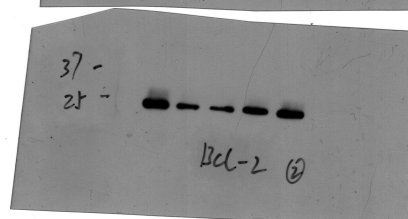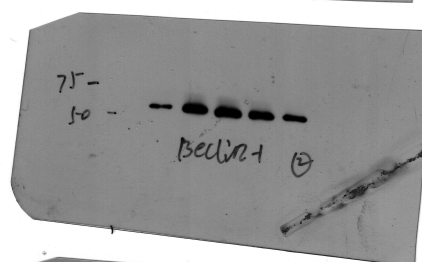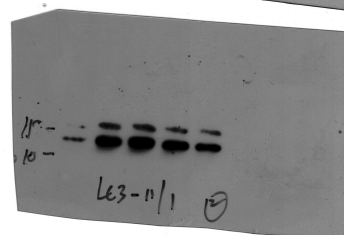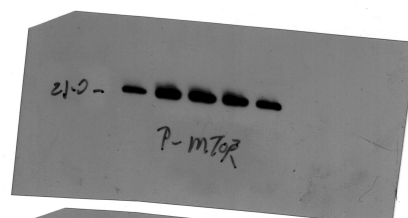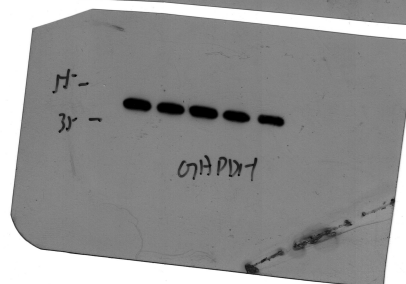

Supplement: Data S1 [file peerj-11-16517-s001.zip › raw data/Figure 5.pdf]

B

Control

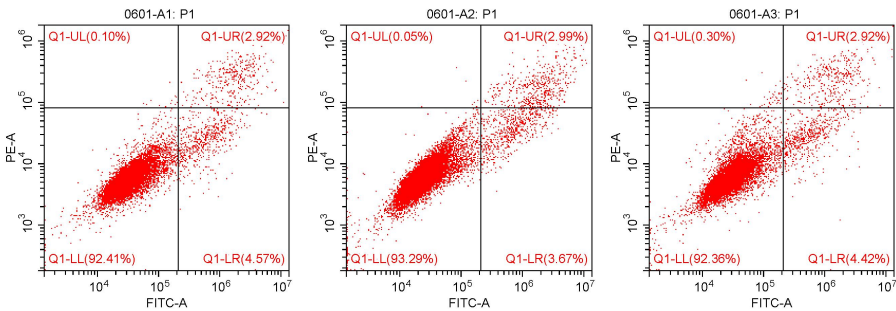

CTX

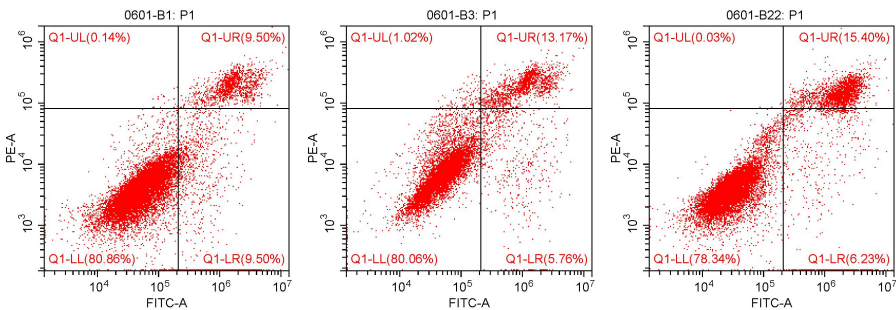

CTX + ADSCs-Exo

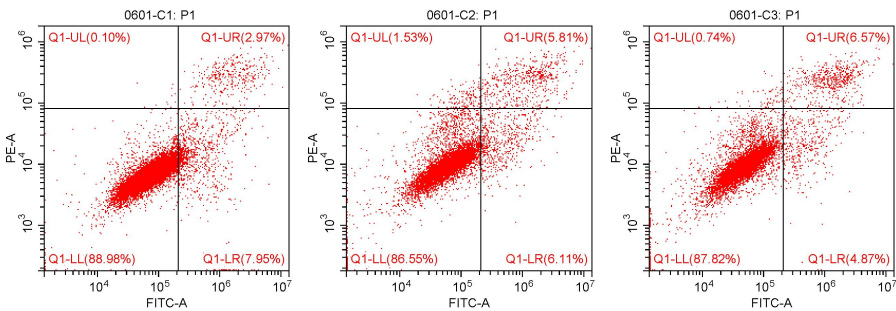

CTX + ADSCs-Exo + Rapa

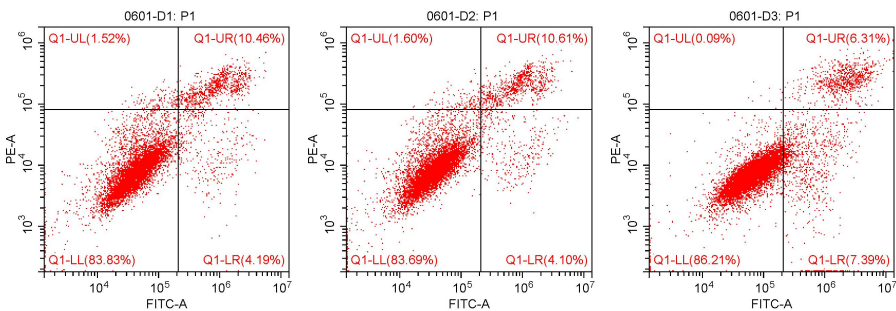

C

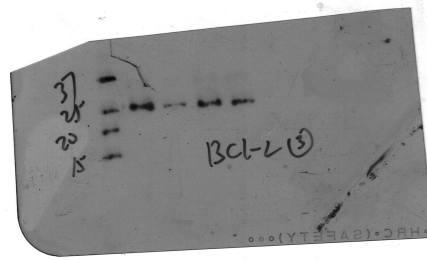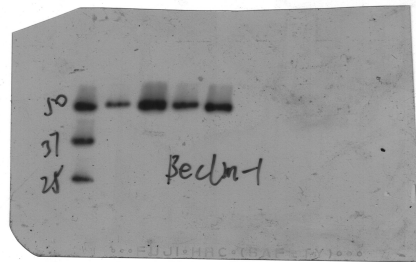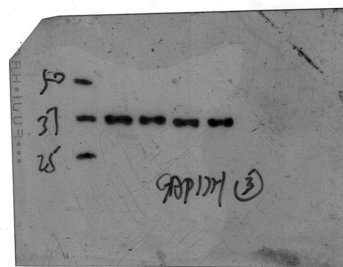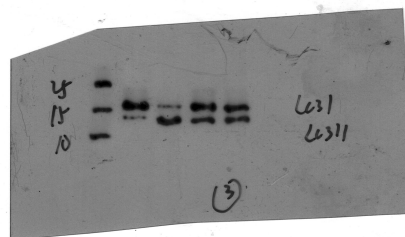

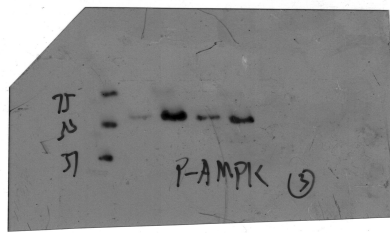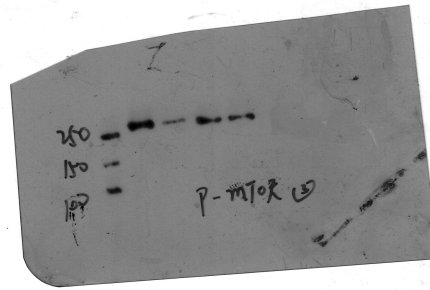

Supplement: Data S1 [file peerj-11-16517-s001.zip › raw data/Figure 6.pdf]

Figure 3

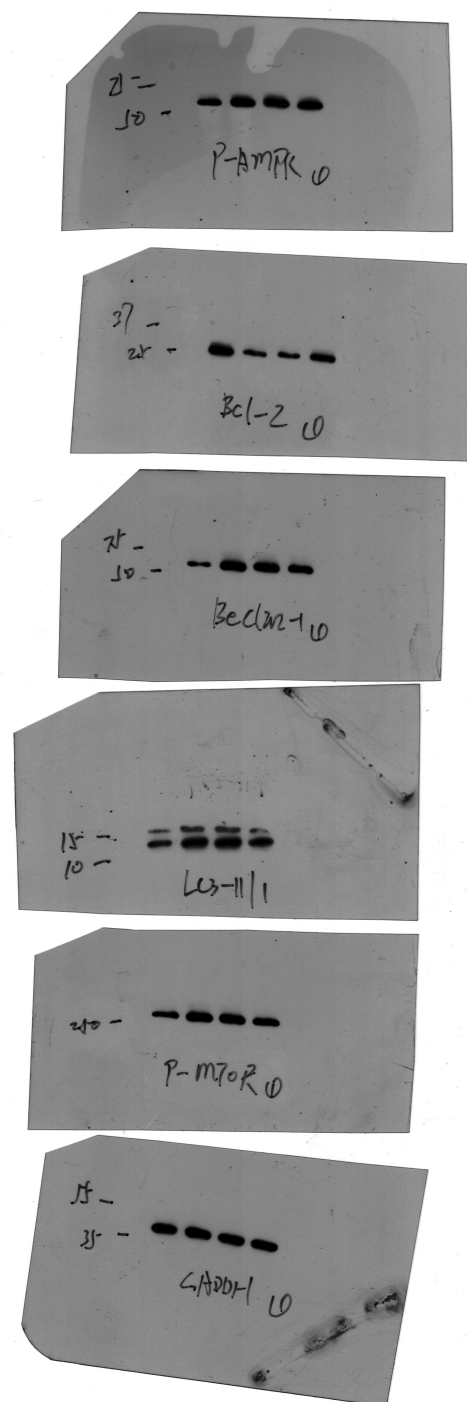

Figure 4B

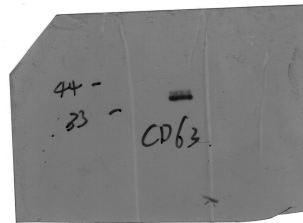

Figure 5C

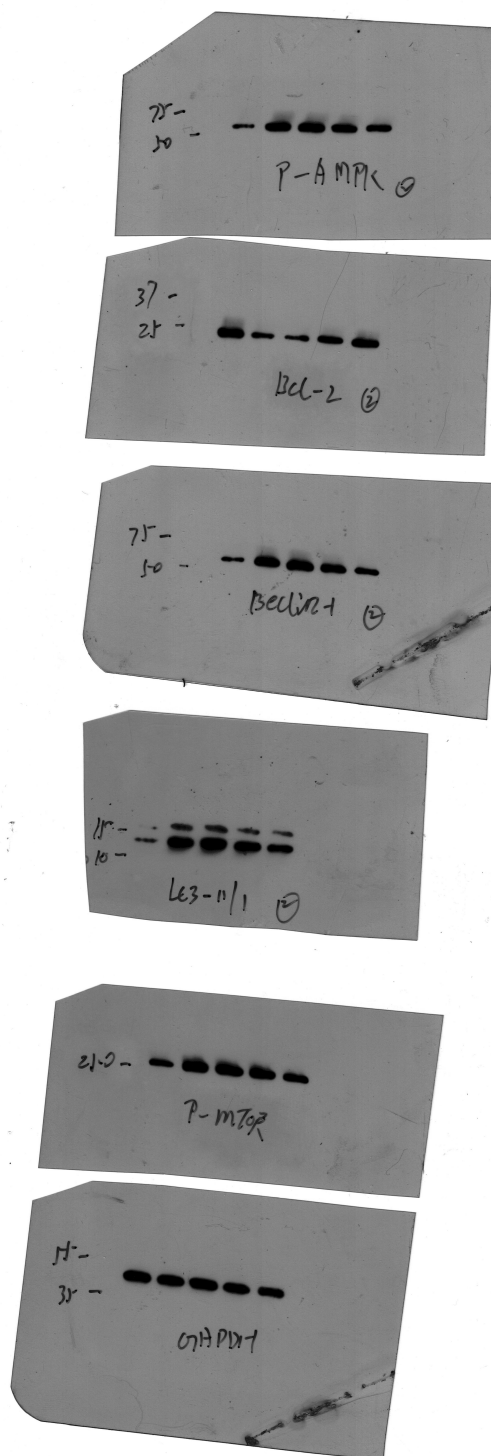

Figure 6C

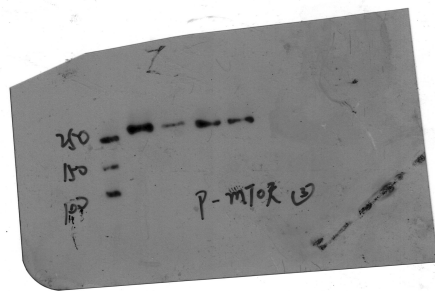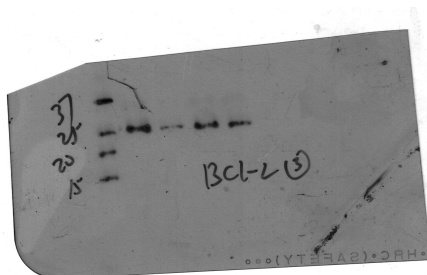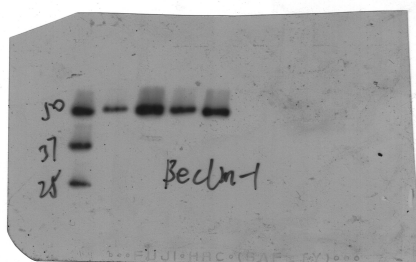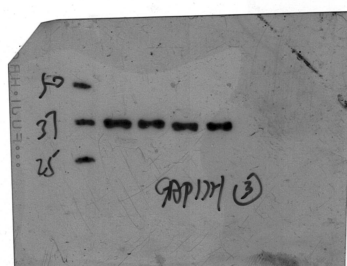

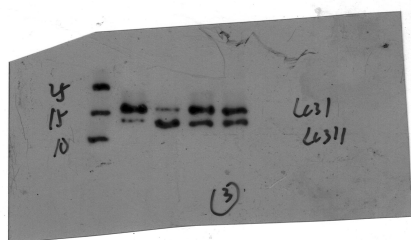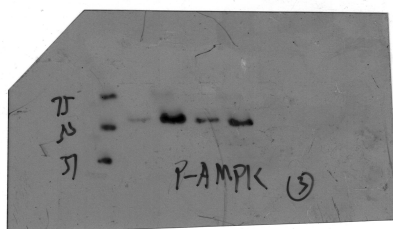

Supplement: Supplemental Information 3 [file peerj-11-16517-s003.pdf]

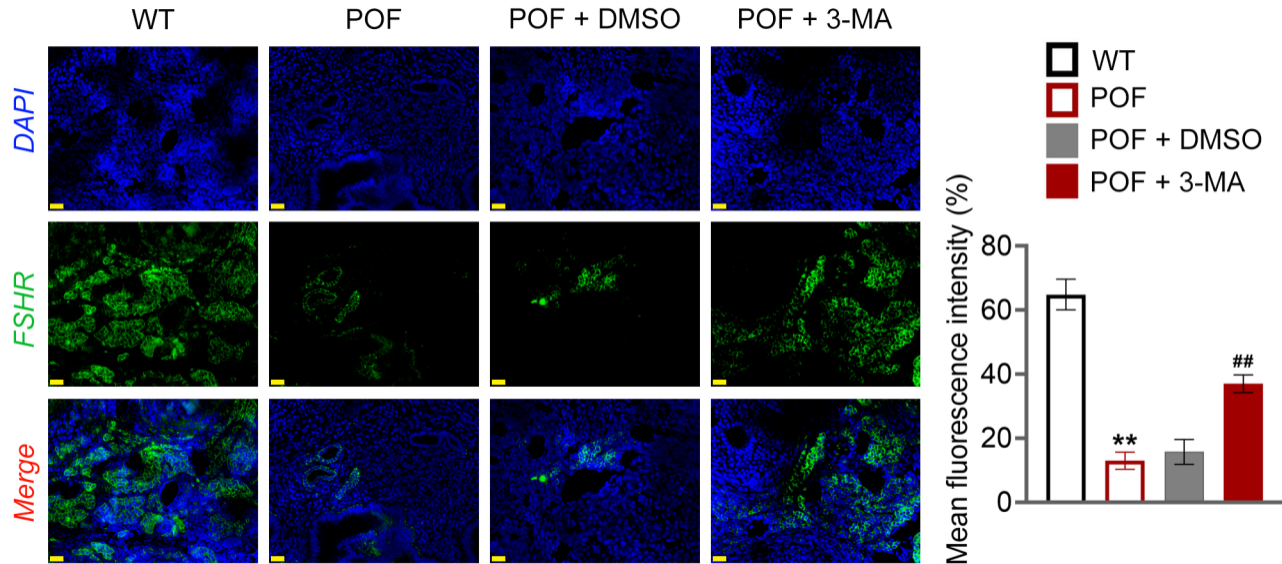

Supplement: Figure S1 — The expression of FSHR was measured by immunofluorescence. Scale bar: 20 µm. ** p < 0.01 compared with WT group, ## p < 0.01 compared with POF + DMSO group. [file peerj-11-16517-s004.pdf]
