# Supplementary material for: Exosomes from adipose-derived stem cells alleviate premature ovarian failure via blockage of autophagy and AMPK/mTOR pathway
Source: PeerJ. 2023 Dec 14;11:e16517. doi: 10.7717/peerj.16517 (PMC10725676; doi:10.7717/peerj.16517)
Supplement: Supplemental Information 2 [file peerj-11-16517-s002.pdf]

# Figure 3C

**Bcl-2**

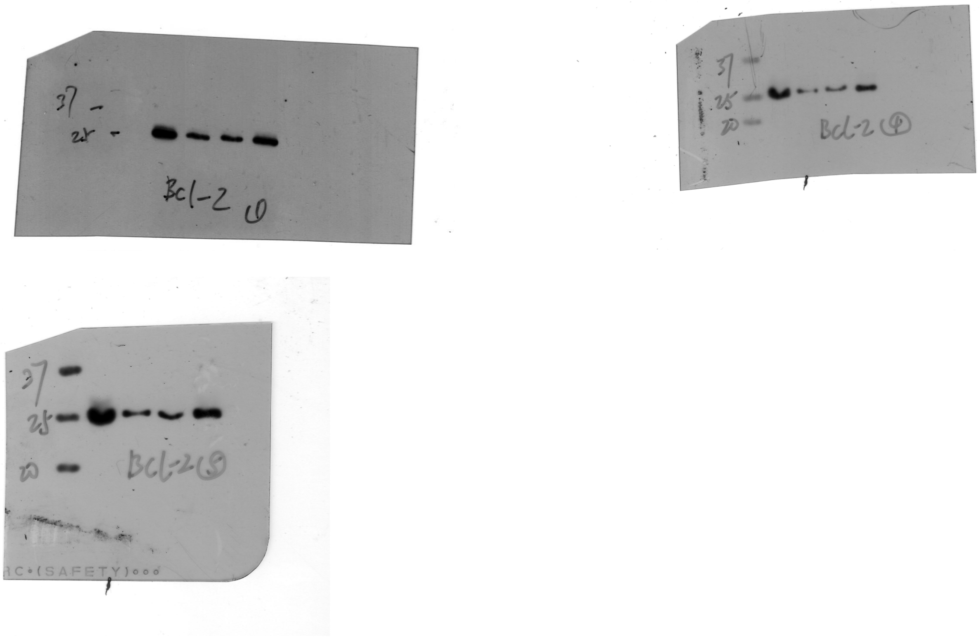

**Beclin-1**

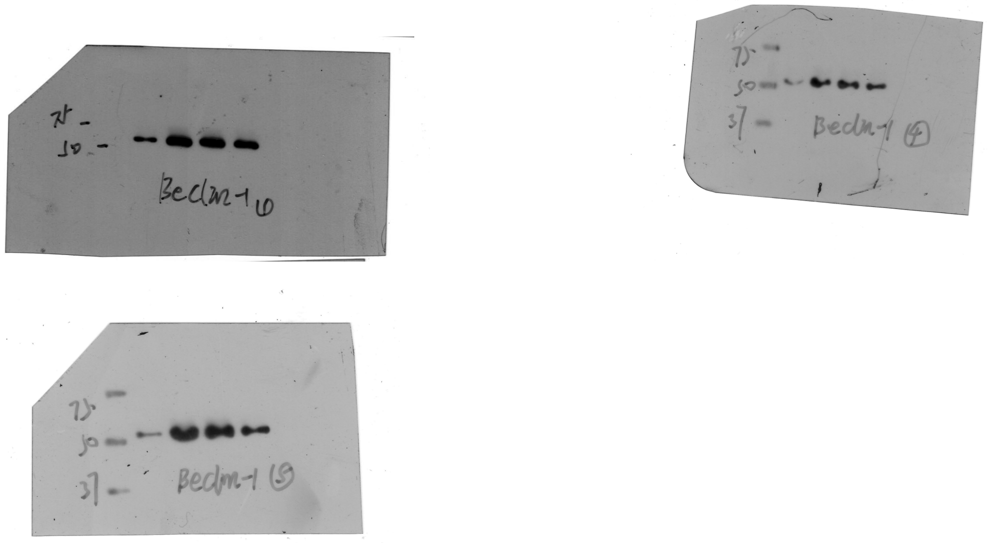

**LC3 I**

**LC3 II**

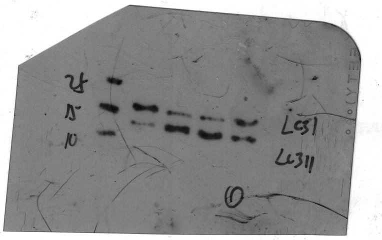
25
15
10
LC3I
LC3II

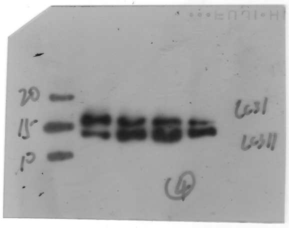
20
15
10
LC3I
LC3II

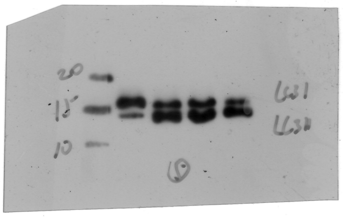
20
15
10
LC3I
LC3II

**AMPK**

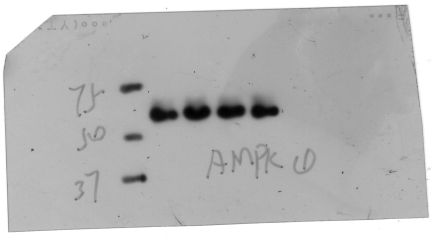
75
50
37
AMPK

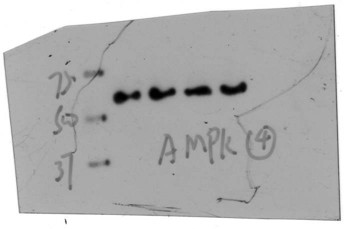
75
50
37
AMPK

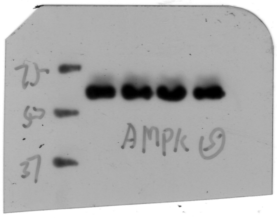
75
50
37
AMPK

**p-AMPK**

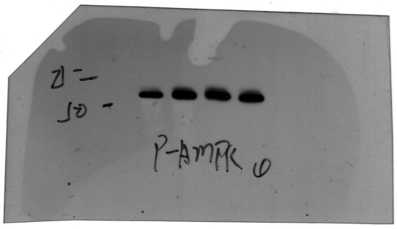
75
50
P-AMPK

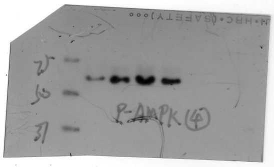
75
50
37
P-AMPK

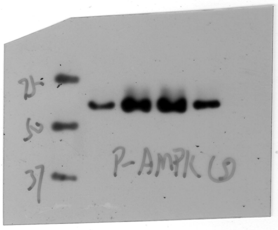

**mTOR**

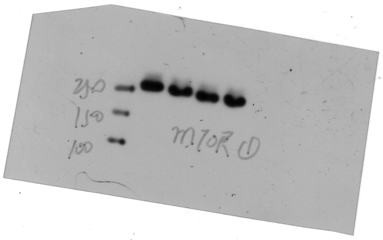

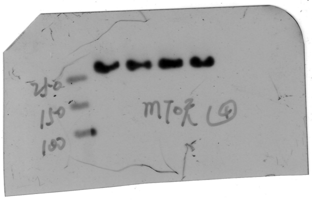

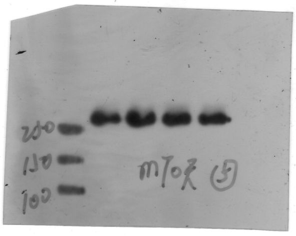

**p-mTOR**

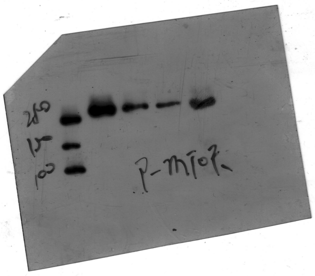

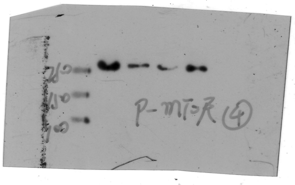

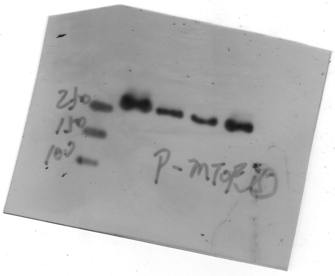

**GAPDH**

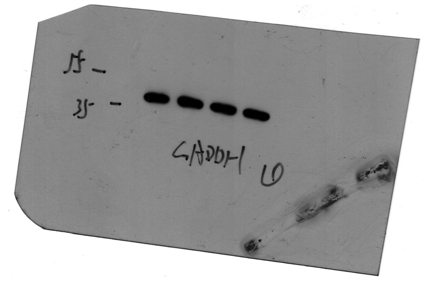


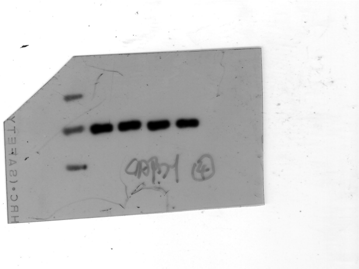

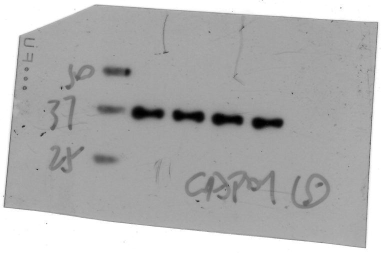

# Figure 4B

**CD63**

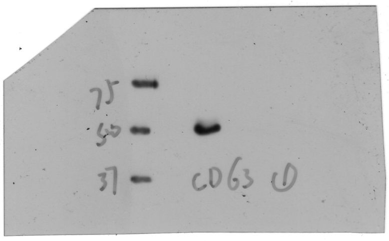


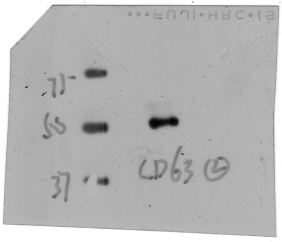


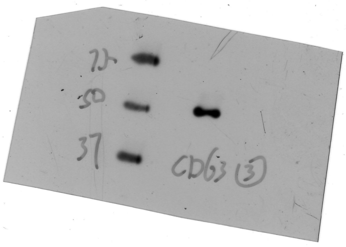


**CD81**

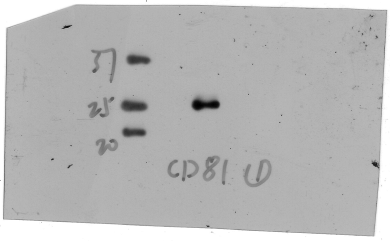


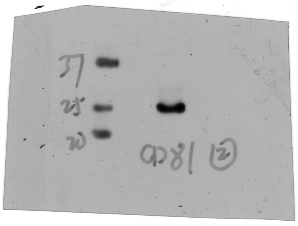


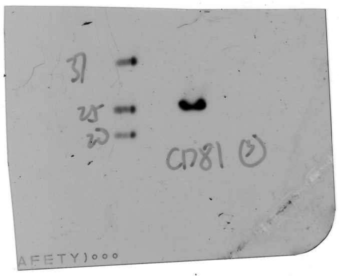


**TSG101**

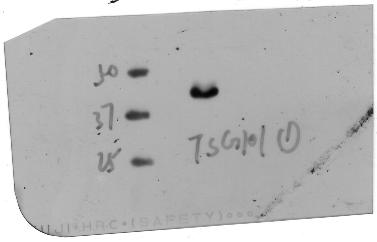


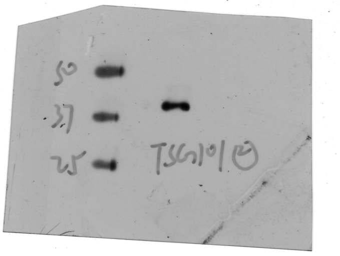


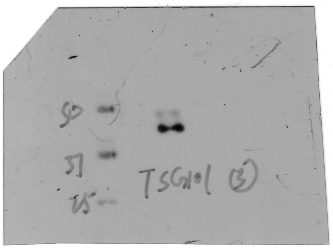


**HSP70**

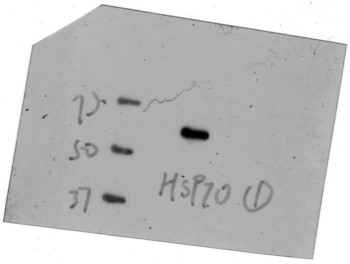


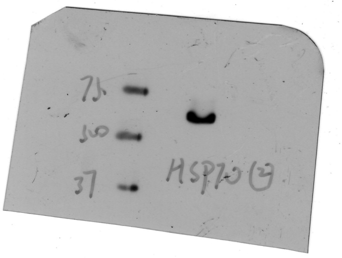


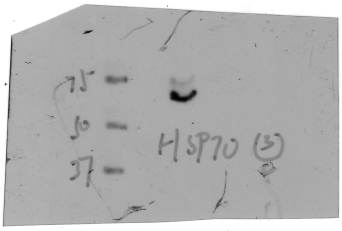


**β-Actin**

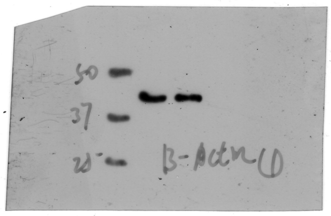


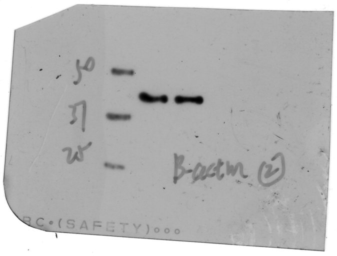

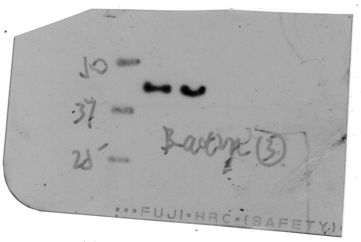
50
37
25
β-actin (3)
FUJI • HRC • (SAFETY)

# Figure 5C

**Bcl-2**

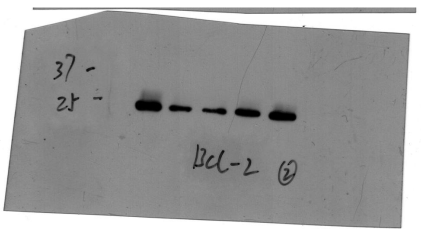


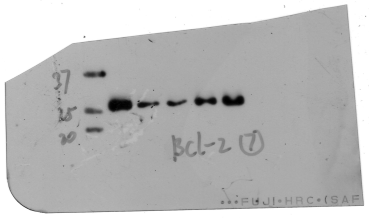


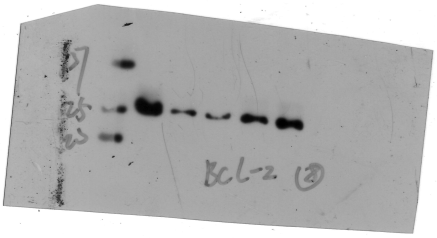


**Beclin-1**

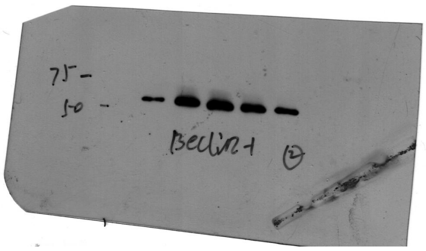


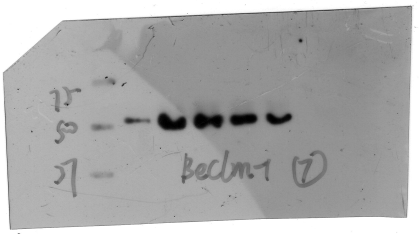


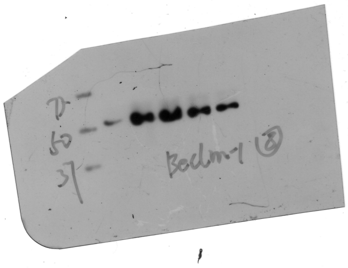


**LC3 I**

**LC3 II**

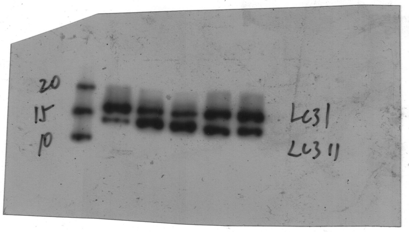
20
15
10
LC3 I
LC3 II

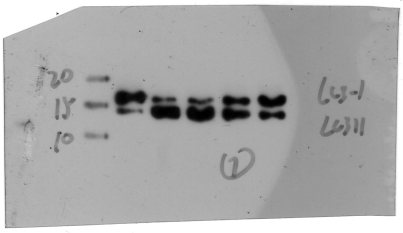
20
15
10
LC3-I
LC3 II

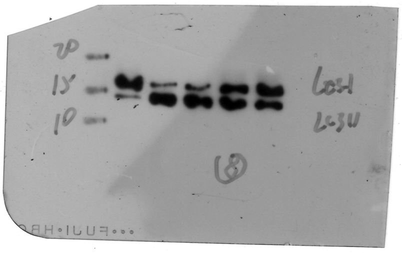
20
15
10
LC3-I
LC3 II

**p-AMPK**

---

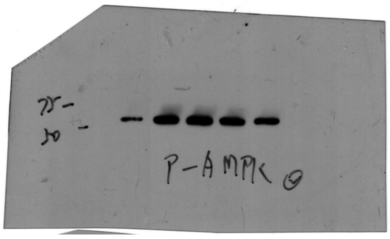
75
50
P-AMPK

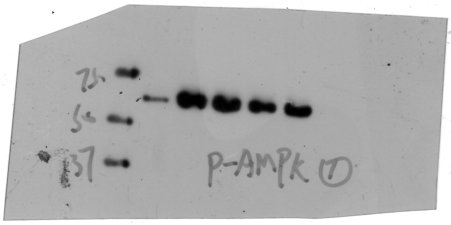
75
50
37
P-AMPK

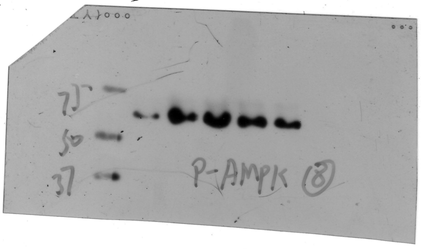
75
50
37
P-AMPK

**p-mTOR**

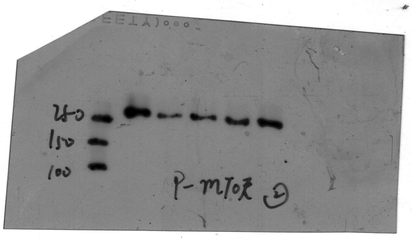
250
150
100
P-mTOR

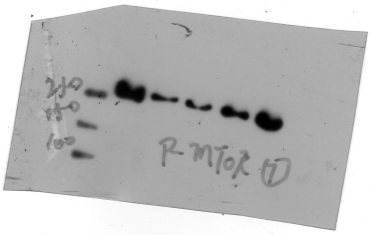
250
150
100
P-mTOR

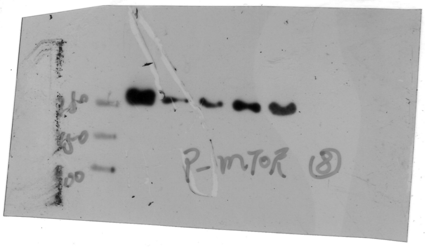


## GAPDH

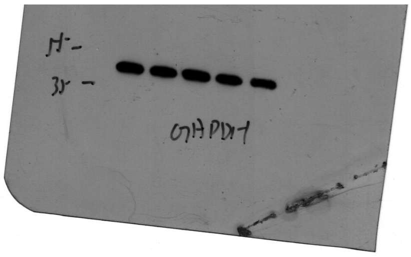


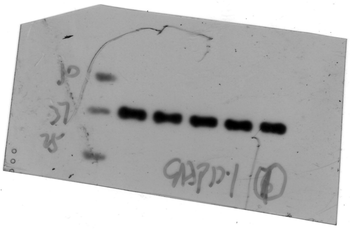

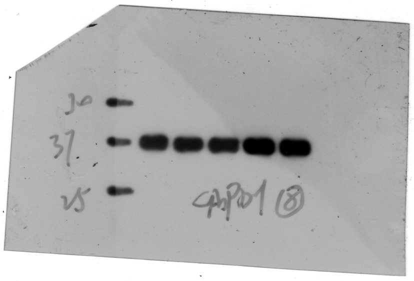

# Figure 6

**AMPK**

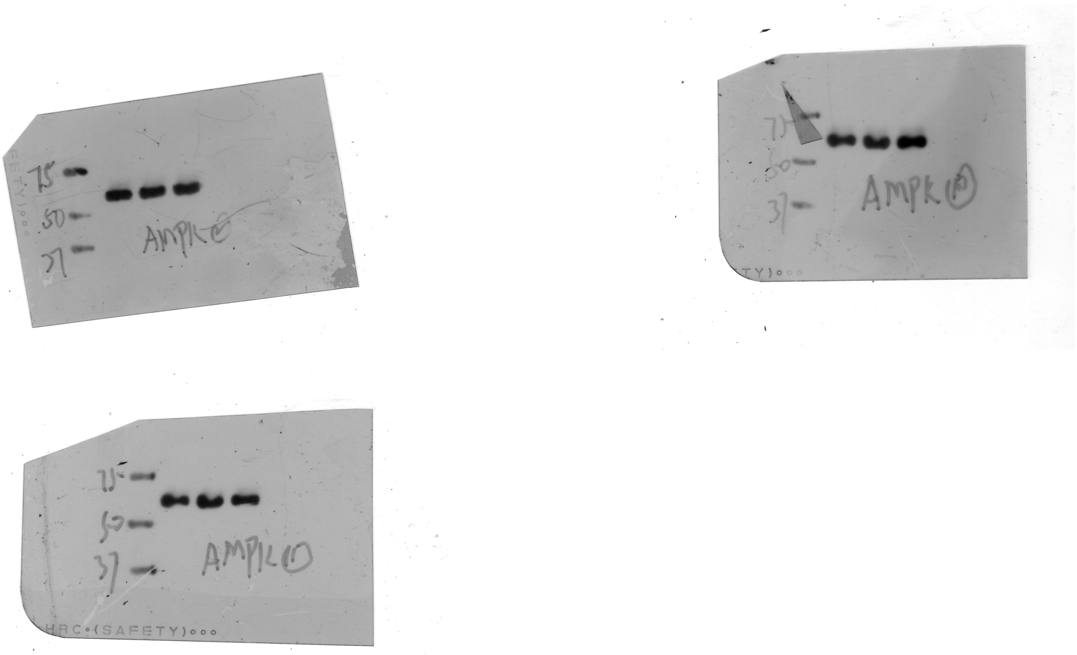


**p-AMPK**

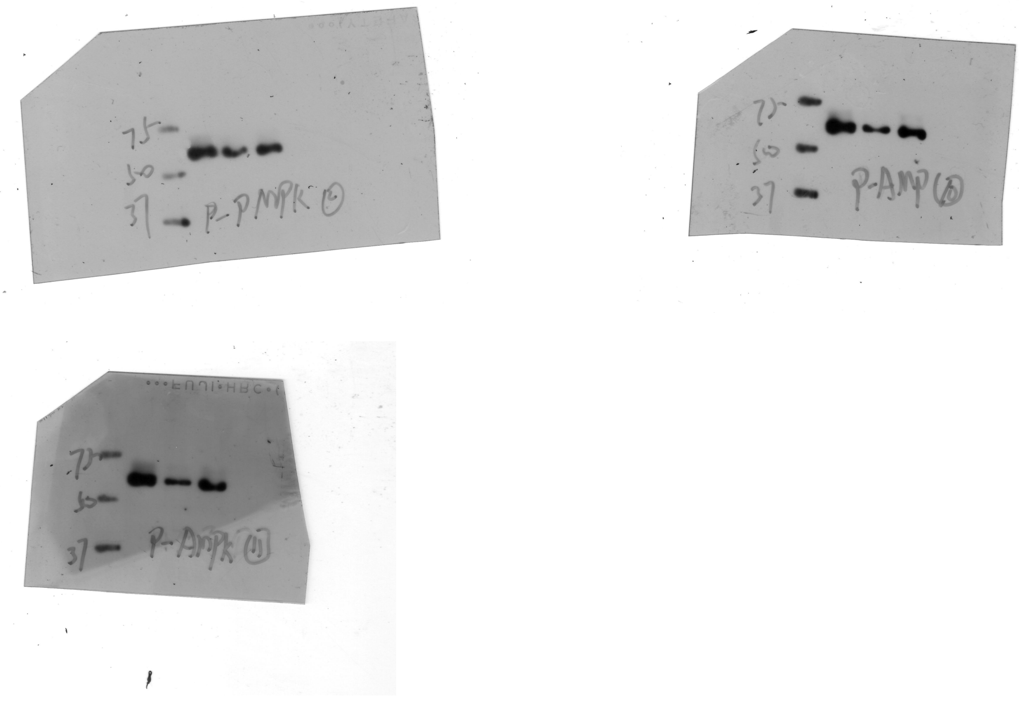


**mTOR**

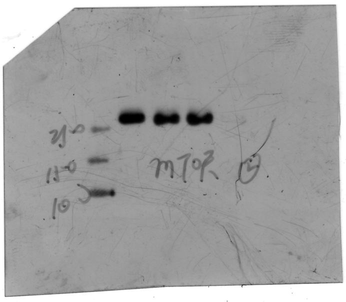

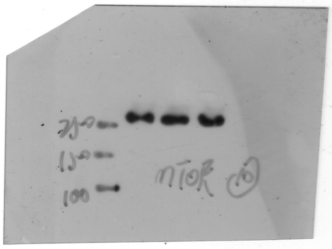

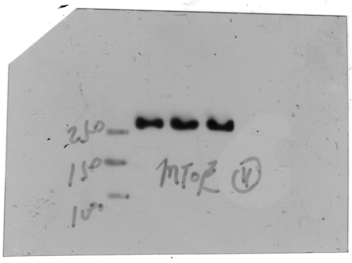

**p-mTOR**

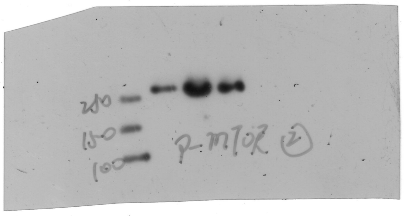

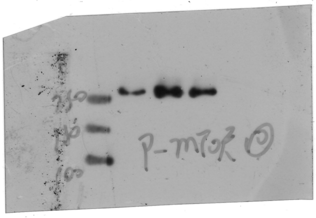

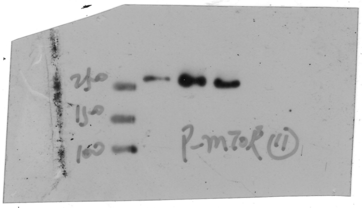

**Bcl-2**

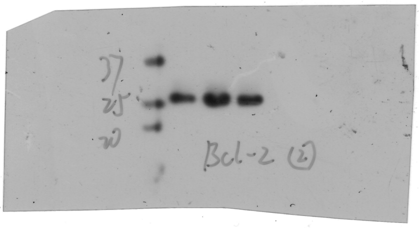

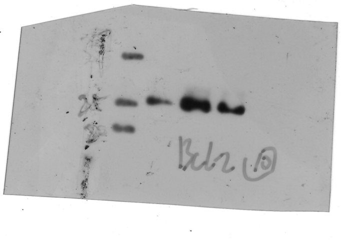

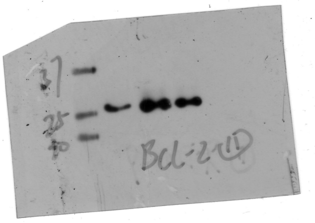

**Beclin-1**

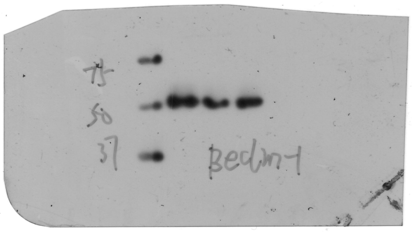

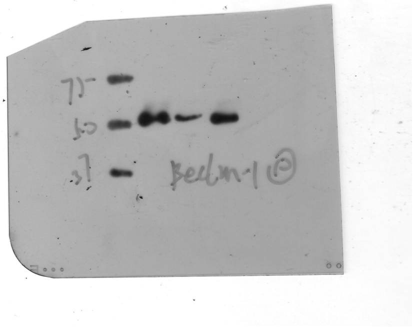

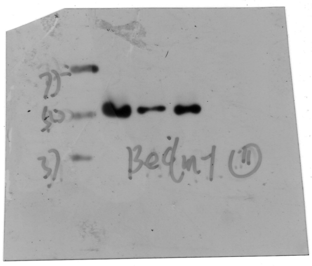

**LC3 I**

**LC3 II**

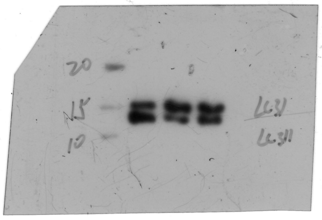

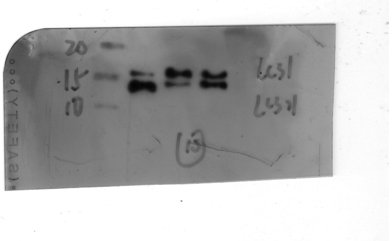

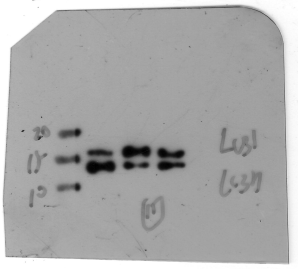

**GAPDH**

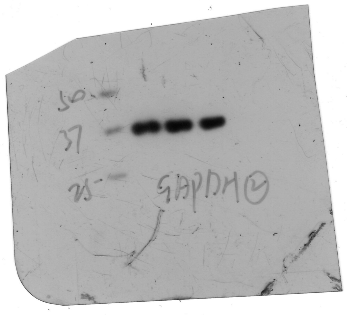


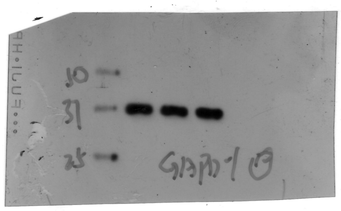

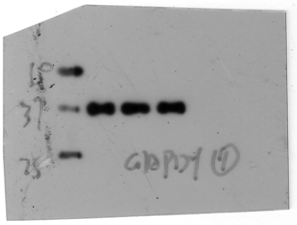

# Figure 8D

**Bcl-2**

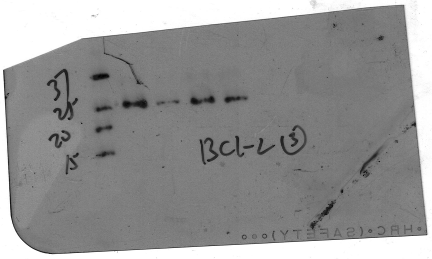

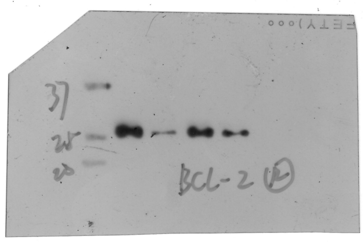

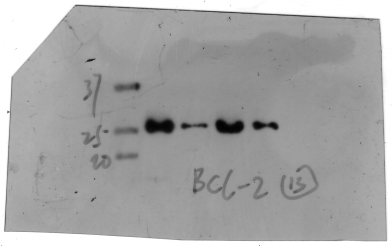

**Beclin-1**

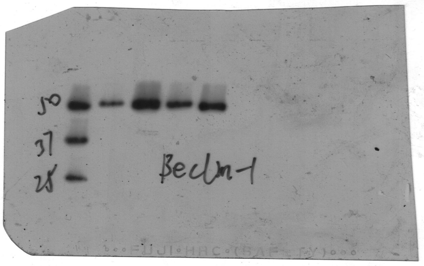

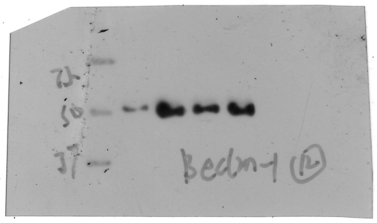

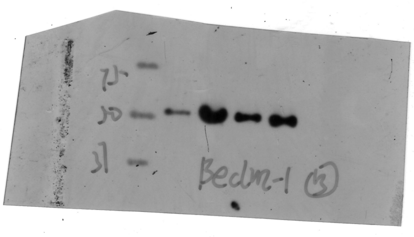

**LC3 I**
**LC3 II**

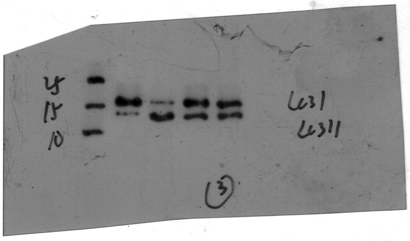
25
15
10
LC3 I
LC3 II
3

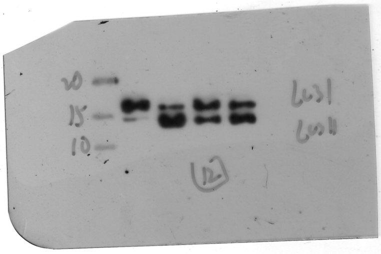
20
15
10
LC3 I
LC3 II
12

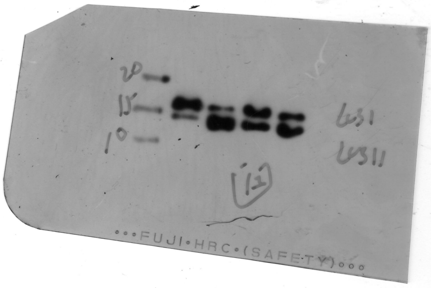
20
15
10
LC3 I
LC3 II
FUJI • HRC • (SAFETY)

**AMPK**

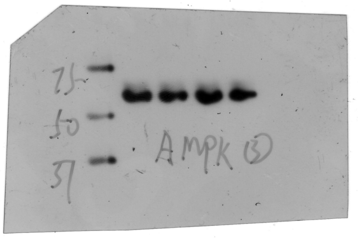
75
50
37
AMPK 3

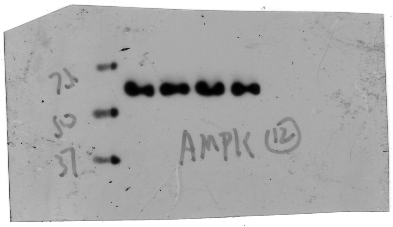
75
50
37
AMPK 12

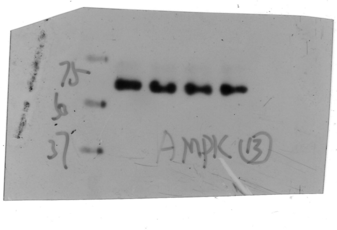
75
50
37
AMPK 13

**p-AMPK**

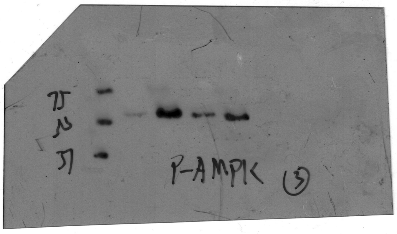
75
50
37
p-AMPK 3

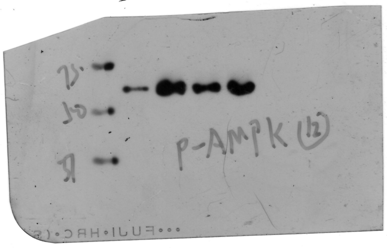
75
50
37
p-AMPK 12

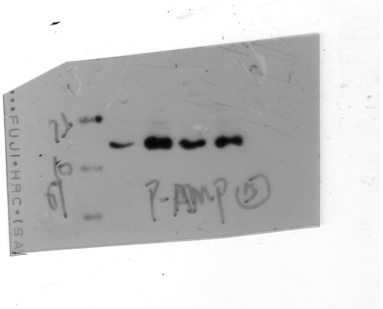

**mTOR**

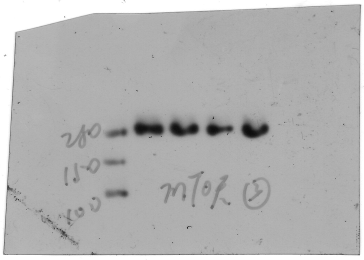

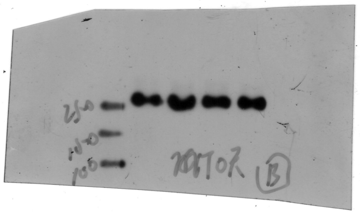

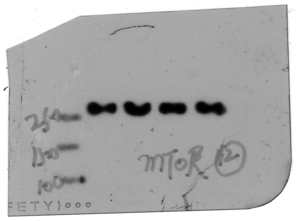

**p-mTOR**

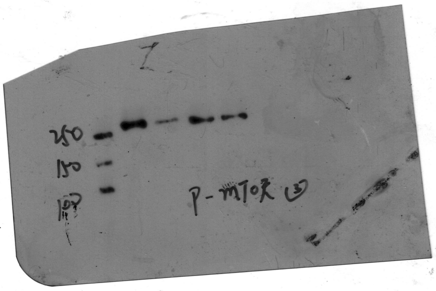

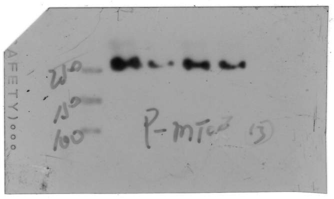

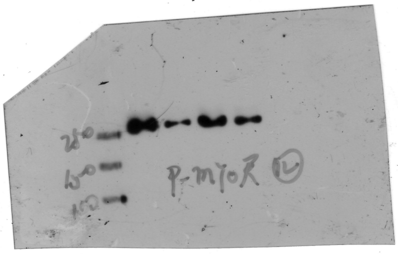

**GAPDH**

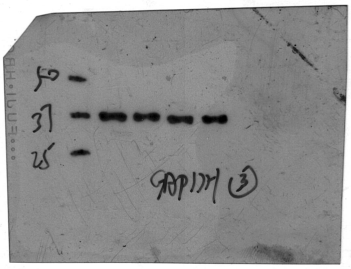
50
37
25
GAPDH

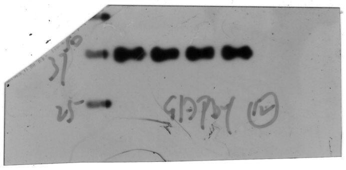
37
25
GAPDH

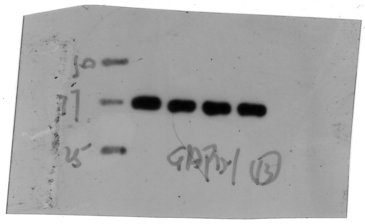
50
37
25
GAPDH
